# Supporting work practices through telehealth: impact on nurses in peripheral regions

**DOI:** 10.1186/1472-6963-11-27

**Published:** 2011-02-04

**Authors:** Marie-Pierre Gagnon, Guy Paré, Hugo Pollender, Julie Duplantie, José Côté, Jean-Paul Fortin, Rita Labadie, Emmanuel Duplàa, Marie-Claude Thifault, François Courcy, Carrie Anna McGinn, Birama Apho Ly, Amélie Trépanier, François-Bernard Malo

**Affiliations:** 1 Research Center of the Centre Hospitalier Universitaire de Québec, Québec, Canada; 2 Faculty of Nursing Sciences, Université Laval, Québec, Canada; 3 Department of Information Technology Management, Hautes Études Commerciales (HEC) Montréal, Canada; 4 Department of Social and Preventive Medicine, Faculty of Medicine, Université Laval, Québec, Canada; 5 Faculty of Nursing Sciences, Université de Montréal, Montréal, Canada; 6 Centre de Santé et de Services sociaux du Grand-Littoral, Lévis, Canada; 7 Faculty of Education, University of Ottawa, Ottawa, Canada; 8 School of Nursing, University of Ottawa, Ottawa, Canada; 9 Department of Psychology, Faculty of Human Sciences, Université de Sherbrooke, Sherbrooke, Canada; 10 Department of Industrial Relations, Faculty of Social Sciences, Université Laval, Québec, Canada

## Abstract

**Background:**

In Canada, workforce shortages in the health care sector constrain the ability of the health care system to meet the needs of its population and of its health care professionals. This issue is of particular importance in peripheral regions of Quebec, where significant inequalities in workforce distribution between regions has lead to acute nursing shortages and increased workloads. Information and communication technologies (ICTs) are innovative solutions that can be used to develop strategies to optimise the use of available resources and to design new nursing work practices. However, current knowledge is still limited about the real impact of ICTs on nursing recruitment and retention. Our aim is to better understand how work practice reorganization, supported by ICTs, and particularly by telehealth, may influence professional, educational, and organizational factors relating to Quebec nurses, notably those working in peripheral regions.

**Methods/Design:**

First, we will conduct a descriptive study on the issue of nursing recruitment. Stratified sampling will be used to select approximately twenty innovative projects relating to the reorganization of work practices based upon ICTs. Semi-structured interviews with key informants will determine professional, educational, and organizational recruitment factors. The results will be used to create a questionnaire which, using a convenience sampling method, will be mailed to 600 third year students and recent graduates of two Quebec university nursing faculties. Descriptive, correlation, and hierarchical regression analyses will be performed to identify factors influencing nursing graduates' intentions to practice in peripheral regions. Secondly, we will conduct five case studies pertaining to the issue of nursing retention. Five ICT projects in semi-urban, rural, and isolated regions have been identified. Qualitative data will be collected through field observation and approximately fifty semi-structured interviews with key stakeholders.

**Discussion:**

Data from both parts of this research project will be jointly analysed using triangulation of researchers, theoretical approaches, methods, and results. Continuous exchanges with decision makers and periodic knowledge transfer activities are planned to facilitate the dissemination and utilization of research results in policies regarding the nursing recruitment and retention.

## Background

### Health care workforce shortages

Workforce shortages in the health care sector are a widespread problem in Canada, as in many countries of the world [1]. This means that the problems related to labour and work places remain a top concern for decision-makers [2]. These problems are particularly apparent in certain areas, such as rural or isolated peripheral regions, due to the significant inequalities in workforce distribution between regions. Canada is the second largest country in the world in terms of territory and, according to international standards, its population is of low density [3]. In fact, about 20% of the Canadian population lives in communities of less than 10 000 inhabitants. Labour shortage in the health care sector is particularly well documented among physicians [4], but also affects other health professionals for which the problem is less documented. These human resources problems are expected to become more problematic in the coming years. In the province of Quebec, the Ministry of Health and Social Services (*French acronym*: MSSS) estimates a current deficit of 3000 nurses, with an expected deficit increase to 10 000 in 2014 and to 17 000 in 2019 [5]. The MSSS recently brought attention to the many symptoms of a profound malaise among care staff in the health system [6]. The use of overtime and mandatory overtime are one of many significant symptoms attesting to the evident difficulties the health system faces in meeting care demands [7]. Furthermore, health care labour shortages constrain the system's ability to respond to the needs of institutions, as well as to those of the population.

In Quebec, the services provided by the health and social service providers in a local health and social services network are coordinated by a local authority, called a Health and Social Services Centre (*French acronym*: CSSS). A CSSS is a multivocational institution operating local community service centres, residential and long-term care centres and, where applicable, a general and specialized hospital centre [8]. The CSSS of many Quebec territories have been facing significant labour problems in the last years. Labour shortage problems are worrisome, due to the possible consequences they may have on access to health services. As well, they exert a direct pressure on existing professionals and can create dissatisfaction related to increased workloads [9]. A study conducted in May 2008 at a semi-urban CSSS reports that the large majority of employees perceived workforce management as *very *or *quite *problematic [10]. As well, the available data on the staff turnover rate paints a worrisome, if not problematic, portrait. For example, over the past years, the semi-urban CSSS Haut-Richelieu-Rouville has faced a constant increase in cancer patients. Other factors, such as acute nursing shortages and recourse to the services of private agencies, have added to the problem. As such, it became crucial for the CSSS to review nursing practices in order to maximise the use of nursing competencies, all while improving the quality and accessibility of client care. A similar situation took place at the semi-urban CSSS Jardins-Roussillon, where a marked increase in home care needs and a continuous decrease in human resources created productivity problems and increased average delays in case management times.

In Quebec, in 2007, the MSSS established a working group on the nursing workforce to find concrete solutions to palliate the problems related to workforce shortages in this area [7]. At the time of their meetings, the members of the working group identified possible reasons that incited nurses to leave the network to join private agencies. Two main reasons were reported: 1) the work hours were more attractive in private agencies; 2) public institutions often had a dysfunctional work organization [7]. It is therefore important and timely to look for innovative solutions, particularly those proposed by the CSSS, and to develop strategies in order to optimise the use of available resources and to design new work practices [2,11].

### ICTs as an innovative solution

In Canada, as in other parts of the world, innovative strategies and solutions are put into place to optimise the use of available resources and to implement new work practices [11,12]. Among these solutions, information and communication technologies (ICTs) are often suggested as having a positive effect on recruitment and retention of staff [13-15]. For instance, according to the MSSS, the health system computerization plan has become an essential tool to reinforce the quality of professional practice and the accessibility of health care and services [16].

ICTs, including telehealth, have the capacity to improve access at all levels of care for a large range of health conditions, offer patient-centred care at lower costs, increase the efficiency of clinical decision-making, and improve the efficacy of chronic health management [17]. These technologies facilitate knowledge acquisition, transfer, and exchange, which can directly impact on many aspects related to the quality of health professionals' professional lives and their levels of work satisfaction [13,15,18]. However, we know little about the impacts of these technologies on the attraction and retention of the health sector workforce in regions with fewer resources. Some studies have nonetheless shown that possibilities for advancement, continued education, and professional development were factors that could influence the retention of medical staff [19-21]. For example, Bernardo *et al*. have demonstrated that their continuing education program (which included ICTs) involving nurses who had left their jobs had an impact on their return to work [22,23]. While certain studies [13,14] show that telehealth has a positive effect on many organizational, professional and educational factors that influence recruitment and retention, current knowledge is still limited about its real impact on recruitment and retention, as well as on health professionals' work [18,24].

In sum, with the increasing implementation of different ICT applications in the Canadian health care system, their impact on work satisfaction and staff retention in the health care sector should be evaluated in a more systematic manner in order to inform health care sector decision-makers [18,24]. Consequently, the present project aims to better understand how work practice reorganization, supported by ICTs, and particularly by telehealth, may influence professional, educational, and organizational factors relating to Quebec nurses, notably those working in peripheral regions.

### Project relevance

The training, planning and distribution of the workforce are among research priorities for health services in Canada [25]. This project corresponds to three of the priority research subject fields identified during the national consultation *Listening for Direction III *on issues related to health services and policies [2]: the workforce and the workplace, change management to improve practices and health, and emerging technologies. It also responds to the needs of decision-makers concerned by the workforce shortage in the health sector [11,26], particularly those of many Quebec CSSS facing the problem of human resources shortages. This project will enable, through the implementation of a solid and continuous partnership between researchers with complementary expertises and decision-makers and stakeholders with extended experience in their milieu, to produce knowledge directly useful for managing health care personnel. There is no doubt that this research directly addresses decision-makers' concerns as well as those of researchers in the area of health services, and is congruent with the objective of the Partnerships for Health System Improvement program, of which this project is part.

### Goals and objectives

The present project aims to better understand the influence of telehealth on professional, educational, and organizational factors concerning nurses working in peripheral regions. According to recent data, these factors could have an influence on nurse recruitment and retention. As well, the project hopes to support the CSSS partners in the evaluation of new work practices related to ICTs and their impact on nurses in Quebec and, more particularly, those working in peripheral regions. This project therefore has two objectives: 1) Study how the use of ICTs in the reorganization of nursing work practices can influence recruitment and retention factors in peripheral regions; 2) Explore how the use of ICTs influences the quality of life at work, the continuity of services as well as continuing education and knowledge transfer opportunities, focusing particularly on five projects that reorganize nursing work practices using ICTs.

### Theoretical framework

Table 1 presents the integrated theoretical framework that identifies telehealth-related factors which could influence the recruitment and retention of health professionals, inspired by the work of Fortin *et al*. [13]. This framework identifies the possible effects of telehealth on many recruitment and retention factors. It presents the effects according to three categories of factors: organizational, professional, and educational.

**Table 1:** Recruitment and retention factors on which telehealth could have an effect

POSITIVE EFFECTS
**Organizational factors**

✓ Counter work overload by (RC & RT):
◦ Putting into place on-call systems
◦ Stabilising services

✓ Increased diversity of available care (RC & RT)

✓ Stimulating work environment (RC & RT)

**Professional factors**

✓ Access to the support of colleagues in large centres (RC & RT)

✓ Decreased feeling of professional isolation (RC & RT)

✓ Possibility to receive a second opinion (RC & RT)

✓ Better knowledge of remote medical teams through distance training (RC)

✓ Contacts between peers (RC & RT)

✓ Discussion about complex cases (RC & RT)

✓ Decision-making support (RC & RT)

**Educational factors**

✓ Increased access to continued education (RC & RT)

✓ Updating knowledge (RT)

✓ Possibility to teach (RC)

✓ Give recruits training better adapted to the needs of remote areas (RT)

**NEGATIVE EFFECTS**

**Organizational factors**

✓ Justify the fact that physicians remain in large centres (RC)

✓ Give perceptions of a staff shortage (RC)

✓ An increase of heavy cases for remote areas (RC)

**Professional factors**

✓ Recruitment of people who do not have the necessary competencies to practice in remote regions (RT)

**Educational factors**

✓ Limited off-site training (RC)

RC = recruitment; RT = retention

The results collected in our previous work [13,14,27,28] allow us use the model of Petterson and Arnetz 1997 [29] to consider the potential influence of telehealth on professional life quality factors. Based on this model, telehealth may influence factors associated with physicians' quality of life at work, through the following effects: 1) support professional competencies and development, by allowing knowledge update and a stimulating work environment; 2) reduce work tension, by facilitating access to a second opinion; 3) improve the organizational climate, through accessibility to the professional support of large centres and to team meetings; and 4) reduce the work load, by putting into place home or regional care systems, and the stabilisation of services. In fact, many of the professionals that were interviewed highlighted the importance of work life quality for retention. In this regard, the most important factor identified by all data sources was workload.

As well, the information gathered by our previous work [13,14,27,28] particularly highlights the potential of telehealth as a knowledge transfer tool. This knowledge transfer, from large centres to remote regions, allows health professionals to increase their access to continued education with the help of distance training. Distance training may also be done from remote regions to large centres, for example for resident training. This may contribute to better understanding remote teams and give potential recruits training that is better adapted to the practical needs of the region.

Lastly, telehealth may have an effect on the three types of continuity of care described by Haggerty *et al*. [30]: informational, relational, and management. Telehealth may allow a specialist to obtain more complete information about his or her patient more quickly, and follow up when visiting the area (or vice-versa). This improves not only efficiency, but also relational continuity. Telehealth may also occasionally ensure certain support services (i.e. pathology) that would allow dependant services (i.e. surgery) to remain in place. It is important to remember that continuity of care reduces certain frustrations that can adversely affect physician retention. As well, we estimate that these findings can be applicable, in part, to the case of nurses working in peripheral regions. In the wake of the introduction of primary health care nurse practitioners (PHCNP) [31], it is important to see how nurses' work and the establishment of the role of PHCNPs may be supported by reorganization projects using ICTs.

## Methods/Design

### General research plan

A participatory research approach was chosen for this research project. This approach allows the issue of nursing workforce to be addressed from its internal social and historical contexts [32]. As such, each step will be taken with decision-makers and project collaborators, in an open and iterative manner, in order to ensure that the results are of use and accurately represent their reality. In doing this, this approach will take into account the needs of each interest group, respecting the specific context of the research [33].

### Descriptive study on the issue of nursing recruitment

To meet the first objective, aiming to better understand how new work practices involving telehealth can influence nursing recruitment, a descriptive study will be carried out of innovative projects in Quebec relating to the reorganization of work practices based upon ICTs. To do this, individual interviews with the Director of Nursing Services (DNS) and Director of Human Resources (DHR) of each CSSS will be undertaken. A general interview framework will guide the interview questions, however the questions may be adapted to the CSSS workforce situation and the nature of the work practice reorganization project. The factors that helped or hindered the recruitment of nurses will be explored. In total, approximately forty interviews will carried out.

However, the number of interviews may vary according to data saturation criterion [34], that is the collection of a full spectrum of opinions. As this study is exploratory and descriptive, we are interested in identifying experiences from different types of regions (urban, peripheral, or remote). The MSSS will initially select among the 95 CSSS in Quebec, to identify those that received financing for an innovative project related to work practice reorganization using ICTs. Stratified sampling will be then used to select approximately twenty CSSS, who will be initially targeted. Next, the DNS and DHR of each selected CSSS will be contacted by the principal investigator and their participation will be solicited.

Before the interview, the participants must read and sign a consent form. The interviews will be approximately one hour in length and will be recorded with the participants' consent. The interviews will be transcribed verbatim and the content will be analysed using N*Vivo software. Thematic content analysis will be done using methods described by Huberman and Miles [35], including codification, organization and treatment. Data codification will be based upon a conceptual model developed in connection with research team's previous work [13,14,27]. The analyses will highlight the similarities and differences between the studied projects according to the different types of regions and technologies used. To ensure the internal validity of the analysis process, the codification of the interviews will be done independently by two of the team's researchers. The results will then be combined and a consensus will be reached as to the final codification. Afterward, the results will be validated with decision-makers and team collaborators.

The results of this first step will allow development of a questionnaire to analyse the influence of telehealth on professional, educational, and organizational factors associated with nursing recruitment as well as factors determining nursing graduates' and newly graduated nurses' intentions to practice in peripheral regions. The study of the intention to practice in a peripheral region will be based upon Ajzen's Theory of Planned Behavior (TPB) [36,37], a model that was previously applied for a similar study of medical residents [28].

The questionnaire development will comply with methodology proposed by Gagné and Godin [38,39]. Adapting the questionnaire to the studied context is an essential research activity to ensure the cultural sensitivity of the study and its validity. For this reason, an anthropological etic-emic approach [40] will be used, as it allows for adapting theoretical constructs to content specific to the studied population. The wording of the questions will follow the theoretical consensus on prediction models [41]. The questions will be created based upon the categories of the systematic review (etic dimension). Qualitative analyses will be done to extract elements to answer each question. Next, the identified themes will allow us to classify the answers gathered. The frequency of each theme will be compiled in order to identify the salient beliefs held by the population (emic dimension). The analysis will be carried out independently by two people. The results will then be compared to identify a final consensus. The identified beliefs will be used to prepare the final questionnaire. The internal consistency of the theoretical constructs and the temporal stability of their measurement will be verified using the test-retest method. To do this, 10 nurses will complete the questionnaire twice in at an interval of two weeks. The internal consistency will be evaluated using Cronbach's alpha [42], and the temporal stability using the intraclass correlation coefficient [43]. A final version of the questionnaire will be produced on the basis of the test-retest results.

The final questionnaire will be distributed to third year students as well as students who graduated during the previous year of from the nursing programs at Université Laval and Université de Montréal. As this is an exploratory study aiming to validate a theoretical model, the goal of the survey is not to generalize the results. As such, convenience sampling among nursing students will be done at the Faculties of Nursing, to which the researchers are affiliated. A letter will be sent to all students to notify them of the study and to solicit their participation by filling out the enclosed questionnaire. The letter will indicate that completing the questionnaire means implied consent to participate in the study. Approximately 600 questionnaires will be distributed and we estimate a response rate of 50%. Consequently, we plan to collect around 300 completed questionnaires, which will allow for adequate statistical significance. In fact, according to Cohen's formulas [44], a sample of 160 is sufficient to detect the effect of two groups of variables on the dependant variable: the first group will formed by three TPB variables (R^2 ^equal to 0.10); the second group will be composed of five additional variables with an increase to R^2 ^equal to 0.02 (alpha = 0.05 and a power of 0.80 for both groups of variables).

The data will be compiled and analysed using SPSS software. First, descriptive analyses of the variables will be done for all variables. Then correlation analyses will be done for theoretical variables. Chi-square analyses will verify the association between the intention to practice in peripheral regions and socioeconomic and educational variables (age, gender, region of origin, spouse's region of origin, type of study program, practicum in a peripheral region) derived from the literature on aspects related to health professional recruitment, forming the control variables. Finally, hierarchical regression analyses will be done to evaluate the influence of the TPB variables on the intention to practice in peripheral regions, as well as the impact of the control variables on this intention. More in-depth analyses (for example, structural equation modeling) could be done according to the relationships discovered between the collected variables.

### Case studies pertaining to the issue of nursing retention

We chose a multiple case study approach, selecting five CSSS in peripheral regions as the studied cases. A case study is an appropriate strategy as it allows us to study a complex phenomenon in a global manner, by situating it in its social, political and historical context [45]. This method has previously been used to analyse the use of research results in decision-making about health services [46]. A case study allows for in-depth analysis of the dynamics associated with the use of telehealth as a new nursing work practice. As well, inter-case allows for rigorous analyses, by allowing for confirmation of hypotheses or the proposal of alternative explanations for a phenomenon [47]. Five CSSS gave their endorsement to the project. The five projects presented below represent the analysed cases.

The first case, at a semi-urban CSSS, has a work practice reorganization project aiming to improve the access to and frequency of services for clients with chronic obstructive pulmonary disease (COPD) by introducing a remote patient monitoring (RPM) project. More precisely, the project seeks to optimise the roles and responsibilities of participants in the continuum of care, in order to maximise work organization. 2- The second case, at another semi-urban CSSS, implanted a work practices reorganization project to support the mobility of home support nurses, including the introduction of a software program, in order to respond to an increased number of cancer patients and an acute shortage of nursing resources. The project, initiated in 2007, principally aimed to introduce a computerized tool for care planning (SyMO) which gives a general overview of clientele profiles, their needs and priority interventions, up to date in real time. This care tool enables the improvement of the quality, the accessibility and the continuity of services, facilitates communication between different professionals, and offers better coordination of interventions, taking into account the rapidly evolving clientele. The new tool facilitates work organization and allows for increased time with clients at home, owing to the streamlining of administrative and secretarial tasks performed by nurses as well as to the availability of an important source of care protocols, facilitating interventions. This pilot project conducted by the Oncology and Palliative Care Unit is now being extended to other sectors of home care within the CSSS. 3- The third case, at a different semi-urban CSSS, currently has a work reorganization project aiming to improve the access of home care services on the basis of the available human, financial, and technological resources available. This is a complex operation of work reorganization based upon the introduction of new computer tools better aligned with new nursing practices (for example, the therapeutic nursing plan). In total, the project concerns more than 100 nurses spread across three establishments. 4- The fourth case study, at a rural CSSS, focuses on a work practices reorganization project aiming to improve the quality and accessibility of health care. The project offers, with the help of telehealth, distance primary health care to the 14 000 residents spread over an immense territory of 19 000 km. In this project, the doctor, who is many kilometres away from the patient, uses the "hands" of a nurse and the "eyes" of adapted instruments. 5- Finally, contacts were established with an isolated CSSS, which has a work practices reorganization project using information technologies to offer distance training for nurses.

Semi-structured interviews with key stakeholders, including regional leaders, managers and nurses, as well as field observation will constitute the principal sources of data in order to well document each case. The respondents will be identified by the contact network method [48] and will be invited to participate in the project. A total of 10 interviews per case are expected, but this number could be lower or higher according to the data saturation criteria [34]. An important element to consider in selecting key contacts will be the representation of a full range of viewpoints, that is the inclusion of participants representing all stakeholders involved in the development of the work reorganization project. Before proceeding to the selection of potential participants, it is necessary to first identify different groups affected directly or indirectly by the introduction of these projects. The chosen participants must read and sign a consent form before the interview. The interviews, approximately one and a half hours in length, will be recorded and transcribed verbatim.

The collected data will be analysed qualitatively using the N*Vivo software. An iterative analysis method will be used as the categories for analysis will first be determined by concepts from our team's previous studies [13,14,27], but could be readjusted in relation to interactions during the course of the research [34]. The first interviews will be coded independently by two researchers and will be presented to the other members of the team to reach a consensus on the final codification method to be used for all interviews. As well, the analysis framework will be validated through a participative process by decision-makers and project collaborators, in order to ensure its pertinence [47] and to encourage stakeholders' ownership of the results and of the research steps [29]. These analyses will identify differences and similarities between cases and highlight the principal effects of ICT introduction on the factors associated with nursing retention in peripheral regions.

### Analyses

The data relating to the two study objectives will be jointly analysed using triangulation of researchers, theoretical approaches, methods, and results [49]. The combination of qualitative and quantitative approaches helps better evaluate the impact that telehealth, as a new work practice, has on factors related to recruitment and retention, contributing to a deeper understanding of the issue [50,51]. The triangulation of the results will build upon the team's previous projects. It will allow identification of professional, educational, and organizational factors related to telehealth that could have an effect on recruitment and retention.

### Linking mechanisms with knowledge users and decision-makers

Knowledge translation (KT) is defined as a continuous exchange process between knowledge producers and users to promote ethical knowledge use [12,52]. According to a review of factors determining knowledge translation in decision-making [53], the two elements most associated with the use of knowledge from research results are the timeliness of the scientific findings and the relationships between the researchers and decision-makers. This is why this project favours an iterative approach, where each research step will be validated by decision-makers to ensure that the research results will be useful for future decision-making [53,54].

With the same goal of ensuring the relevance of this study and the use of its findings [55], the team is formed of decision-makers, researchers, and collaborators (knowledge users). The relationships between the team members are already established. Two one-day meetings were held in order to determine decision-makers' needs regarding the research and to establish partnerships between decision-makers and researchers. Incidentally, the majority of the decision-makers involved in this project have already collaborated with a number of other projects involving the team's researchers.

Six other meetings between researchers, decision-makers, and users are anticipated throughout the project [56]. The results of each step of the research will be discussed at these meetings to ensure understanding and pertinence for decision-makers. As well, the meetings will allow for the research activities to be adjusted according to the needs and comments of decision-makers and collaborators. The meetings will encourage commitment to and ownership of the research results. Finally, a monthly report will be sent by email to all members of the team, including decision-makers, in order to keep them up to date on the project's advancement and any changes to its progress.

It is important to note that the research team was established to ensure the relevance of the study and the use of its results [55]. As such, its members include decision-makers and knowledge users directly involved in the search for solutions to workforce-related problems.

### Knowledge translation plan

The end-of-grant knowledge translation of the project has three objectives: 1) Ensure that our findings and recommendations correspond to the needs of local decision-makers and to the realities of health professionals; 2) Allow for the use of our results and recommendations by decision-makers across the province of Quebec and eventually in other Canadian jurisdictions; 3) Have our results validated by the international scientific community.

For the first objective, the intended audience will be the health care professional associations, the CEOs and the DHRs of health care organisations in the region. The participation of decision-makers in the dissemination of the results at this level is essential as many studies have shown the importance of key stakeholders in knowledge translation [56]. The results will also be shared at meeting with representatives of the professional associations and unions of the CSSS that are part of the project. These meetings are important because the union representatives are particularly sensitive to labour issues. Also, these meetings aim to be interactive in order to ensure that the recommendations take into account the perceptions of groups representing health professionals. Following these meetings, key messages will be sent to the newspapers of different professional associations and unions. As well, a presentation of the results and recommendations will be done at a meeting of the Regional Table of CEOs and DHR.

For the second objective, involving decision-makers across the province of Quebec and of Canada, activities will first target the Health and Social Services Agencies (*French acronym*: ASSS) of Quebec. A non-technical language report which follows the 1-3-25 format, considered an efficient way to reach decision-makers [57], will be sent to the DHR and CEO of each ASSS and the MSSS. After this mailing, we will invite the Directors of each ASSS and the MSSS to meet with the team in order to present to them the results and recommendations. As well, results will be presented at events involving clinicians and managers. In order to reach Canadian decision-makers, key messages will be translated and sent to different Canadian newspapers and we will prepare information letters for specific audiences, such as the newsletter of the Canadian Medical Association.

Lastly, for the third objective of the knowledge translation plan, the results will be published in scientific journals with high impact factors. The results will be presented at the annual congress of the Latin Association for Health Systems Analysis, where a session on the workforce and ICTs will be proposed. To ensure that the three KT objectives are reached, a short questionnaire on the quality of the presentations and the intentions to use the results will be distributed to attendees at the end of each presentation. This questionnaire will be adapted from psychosocial theories to explain knowledge use behaviour [37,58]. The results of this evaluation will allow adjustment of the KT plan according to the needs of different audiences.

## Competing interests

The authors declare that they have no competing interests.

## Authors' contributions

MPG, HP, and JD drafted the study protocol. CAM translated and adapted the protocol for publication. All authors critically revised and approved the final manuscript.

## Pre-publication history

The pre-publication history for this paper can be accessed here:

http://www.biomedcentral.com/1472-6963/11/27/prepub
